# Midnolin, a Genetic Risk Factor for Parkinson’s Disease, Promotes Neurite Outgrowth Accompanied by Early Growth Response 1 Activation in PC12 Cells

**DOI:** 10.1080/10985549.2024.2399358

**Published:** 2024-09-12

**Authors:** Ayano Chiba, Chisato Kato, Tadashi Nakagawa, Tsukasa Osaki, Kohei Nakamura, Ikuo Norota, Mikako Nagashima, Toru Hosoi, Kuniaki Ishii, Yutaro Obara

**Affiliations:** a Department of Pharmacology, Yamagata University School of Medicine, Yamagata, Japan; b Department of Clinical Pharmacology, Faculty of Pharmaceutical Sciences, Sanyo-Onoda City University, Sanyo Onoda, Japan; c Department of Biochemistry and Molecular Biology, Yamagata University School of Medicine, Yamagata, Japan

**Keywords:** PC12 cells, Parkinson’s disease (PD), early growth response 1 (EGR1), neurofilament light chain (NEFL), neurite outgrowth

## Abstract

Parkinson’s disease (PD) is an age-related progressive neurodegenerative disease. Previously, we identified midnolin (*MIDN*) as a genetic risk factor for PD. Although *MIDN* copy number loss increases the risk of PD, the molecular function of MIDN remains unclear. To investigate the role of MIDN in PD, we established monoclonal *Midn* knockout (KO) PC12 cell models. *Midn* KO inhibited neurite outgrowth and neurofilament light chain (*Nefl*) gene expression. Although MIDN is mainly localized in the nucleus, it does not encode DNA-binding domains. We therefore hypothesized that MIDN might bind to certain transcription factors and regulate gene expression. Of the candidate transcription factors, we focused on early growth response 1 (EGR1) because it is required for neurite outgrowth and its target genes are downregulated by *Midn* KO. An interaction between MIDN and EGR1 was confirmed by immunoprecipitation. Surprisingly, although EGR1 protein levels were significantly increased in *Midn* KO cells, the binding of EGR1 to the *Nefl* promoter and resulting transcriptional activity were downregulated as measured by luciferase assay and chromatin immunoprecipitation quantitative real-time polymerase chain reaction. Overall, we identified the MIDN-dependent regulation of EGR1 function. This mechanism may be an underlying reason for the neurite outgrowth defects of *Midn* KO PC12 cells.

## Introduction

Parkinson’s disease (PD) is the second most common neurodegenerative disease. Motor impairments are a major symptom of PD, and result from a loss of dopaminergic neurons in the substantia nigra compacta of the midbrain that project to the striatum. The number of PD patients is growing faster than that of any other neurological disorder.^1^ A better understanding of the root causes of PD and highly effective therapies are therefore needed.^2^ Genetic, epigenetic, and environmental factors affect both the onset and progression of PD in complex ways. Although most cases are idiopathic, approximately 10% of PD cases are familial, and more than 20 causative genes with high penetrance have been identified including synuclein alpha (*SNCA*), parkin RBR E3 ubiquitin protein ligase (*PRKN*), and leucine rich repeat kinase 2 (*LRRK2*).^3–6^ In addition, glucosylceramidase beta 1 (*GBA1*) is one of the largest genetic risk factors, with higher frequency and lower penetrance than causative genes.^4^

Midnolin (*MIDN*) was originally identified in embryonic stem cells.^7^ It is abundantly but selectively expressed in the midbrain at early developmental stages (embryonic days 10.5–12.5) and is distributed more widely at neonatal and adult stages. Although it was originally reported as a nucleolar localizing protein,^7^ more recent reports suggest that its intracellular localization is mainly in the nucleus.^8^^,^^9^ In neuronal cell lines, including PC12 cells and SH-SY5Y cells, *MIDN* expression is reportedly induced by growth factors including nerve growth factor (NGF), brain-derived neurotrophic factor (BDNF), and insulin; this is accompanied by the activation of extracellular signal-regulated kinase (ERK)1/2, ERK5, and phosphoinositide 3-kinase (PI3K).^9^^,^^10^ *Midn* mRNA can also be induced by potassium chloride depolarization and serum stimulation in primary cortical neurons and NIH3T3 fibroblasts, respectively.^11^ Furthermore, upregulated MIDN has been reported to enhance ubiquitin-independent proteasomal degradation.^11^^,^^12^

Regarding the association between *MIDN* and disease, a *MIDN* variant has been identified as a candidate causative variant for autism spectrum disorder.^13^ In addition, duplications in 19p13.3, including the *MIDN* locus, are associated with male infertility.^14^ Recently, *MIDN* has also been reported to promote proliferation and tumor formation in liver cancer cells,^15^ and the severity of nonalcoholic fatty liver disease is attenuated in heterozygous *Midn* knockout (KO) mice.^16^ Furthermore, MIDN is essential for B cell leukemia malignancies.^17^ However, the molecular pathogenetic role of MIDN in these diseases remains unclear. Previously, we have identified *MIDN* as a potent genetic risk factor for PD in both Yamagata (Japan) and British cohorts.^9^^,^^18^^,^^19^ Copy number loss (deletion) of *MIDN* was observed in 10.5% (9 of 86) and 6.55% (142 of 2168) of patients with sporadic PD in Yamagata and Britain, respectively. By contrast, we identified a lower frequency of *MIDN* loss in other populations; *MIDN* loss was identified in 0% (0 of 100) of healthy controls and 0.0662% (2 of 3021) of the general population in Yamagata, and in 1.64% (47 of 2860) of the general population in Britain.

We have previously demonstrated that MIDN is responsible for PD-associated phenotypes such as neurite outgrowth and PRKN expression in PC12 cells.^9^^,^^20^ Nevertheless, the molecular entity of MIDN that regulates neuronal phenotypes remains unclear. Using transcriptome analysis, we revealed that MIDN affects the expression of a wide variety of genes.^20^ However, MIDN itself is assumed to not be a typical transcription factor because it does not encode DNA-binding motifs or other transactivation domains, although it does encode an ubiquitin-like domain and nuclear/nucleolar localization signal. We therefore hypothesized that MIDN may bind to and regulate certain transcription factor(s). In the present study, we conducted an in silico analysis of RNA sequencing (RNA-seq) data, and identified that the consensus sequences of at least four transcription factors—early growth response 1 (EGR1), SMAD family member 3 (SMAD3), GATA binding protein 3 (GATA3), and PLAG1 zinc finger (PLAG1)—are shared in the promoter regions of downregulated genes in MIDN-deficient cells. Among these factors, EGR1 is an immediate–early gene that is rapidly and robustly induced by various stimuli including NGF and is essential for neurite outgrowth in PC12 cells.^21^^,^^22^ We therefore hypothesized that EGR1 may be one of the binding partners of MIDN that is regulated by MIDN. Thus, in the present study, we aimed to examine the regulation of EGR1 activity by MIDN.

## Results

### *Midn* is required for neurite outgrowth in PC12 cells

**Figure 1.**
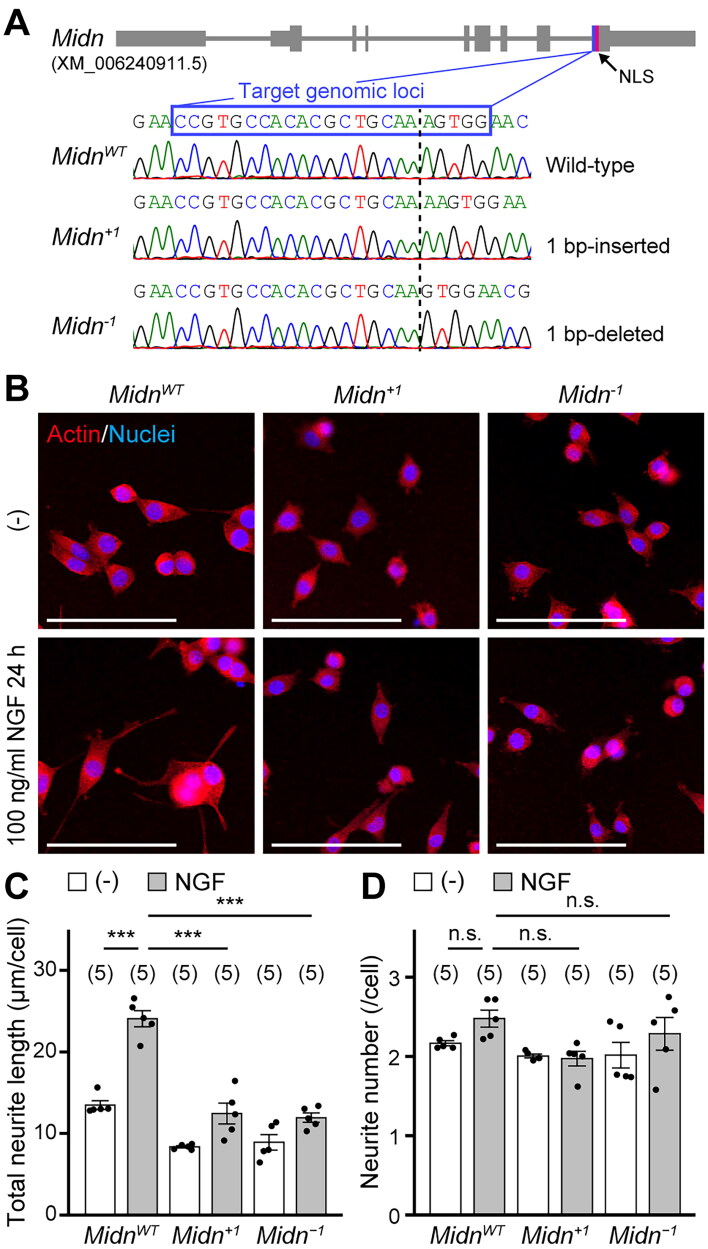
Midnolin (MIDN) is responsible for neurite outgrowth in PC12 cells. (A) *Midn* cDNA sequences from wild-type (WT) PC12 cells and monoclonal *Midn* knockout (KO) PC12 cells established using clustered regularly interspaced short palindromic repeats (CRISPR)/CRISPR-associated protein 9 (Cas9)-mediated genome editing. The gray lines at the top indicate the *Midn* gene. Bold, medium, and thin lines indicate the coding sequence, untranslated regions, and introns, respectively. The *Midn* frameshift mutation was homozygously induced near the nuclear localization signal (NLS). Dotted lines indicate inserted or deleted sites. *Midn*^WT^, a WT clone; *Midn^+1^*, a single nucleotide-inserted clone; *Midn^−1^*, a single nucleotide-deleted clone. (B) Representative images of *Midn*^WT^, *Midn*^+1^, or *Midn*^−1^ PC12 cells treated with or without nerve growth factor (NGF, 100 ng/mL, 24 h). Actin fibers and nuclei were stained with rhodamine-phalloidin (5 U/mL) and Hoechst-33258 (1 μg/mL), respectively. NGF-induced neurite outgrowth was reduced in *Midn* KO PC12 cells. (–), Absence of NGF; scale bars, 100 μm. (C) Quantitative analysis of the total neurite length shown in (B), measured as described in the Materials and Methods. In this and the following graphs, unless otherwise noted, each dot represents a single sample value; the number of samples is indicated at the top. (D) Quantitative analysis of the number of neurites shown in (B), measured as described in the Materials and Methods. n.s., not significant; ***P < 0.001 (Tukey’s test). Data are expressed as the mean ± standard error.

To investigate the molecular function of MIDN, we have previously disrupted *Midn* expression in PC12 cells using clustered regularly interspaced short palindromic repeats (CRISPR)/CRISPR-associated protein 9 (Cas9)-mediated frameshift mutation and a premature termination codon.^9^ Herein, we refer to these mutant PC12 cells as *Midn* KO polyclonal PC12 cells. We used the PC12 rat adrenal pheochromocytoma cell line as a model to study neuronal cell biology because these cells exhibit some aspects of the dopaminergic neuronal phenotype. To further exaggerate the *Midn* KO phenotype, we cloned and established monoclonal PC12 cell lines from the previously established *Midn* KO polyclonal PC12 cells. In the monoclonal PC12 cell lines, the *Midn* frameshift mutation was homozygously induced (*Midn^+1^*, a single nucleotide-inserted clone, and *Midn^−1^*, a single nucleotide-deleted clone) near the nuclear localization signal (Figure 1A). We confirmed the reduction of *Midn* mRNA expression in *Midn* KO PC12 cells compared with wild-type (WT) PC12 cells (*Midn*^WT^) (Figure S1A). In addition, we validated these cell lines by observing total neurite outgrowth with and without NGF incubation (100 ng/mL, 24 h) (Figure 1B). As expected, NGF-induced neurite outgrowth was completely inhibited in *Midn* KO PC12 cells compared with *Midn*^WT^ cells, indicating that *Midn* is required for neurite outgrowth. Although neurite length (μm/cell) was significantly reduced in *Midn* KO PC12 cells (Figure 1C) compared with *Midn*^WT^ cells, the number of neurites per cell was not reduced (Figure 1D). The monoclonal *Midn* KO PC12 cell lines exhibited a similar neurite outgrowth defect phenotype to the polyclonal *Midn* KO PC12 cells.^9^ We therefore focused on the regulatory mechanisms of neurite outgrowth in these cells to elucidate the molecular function of MIDN.

### MIDN regulates neurofilament expression

**Figure 2.**
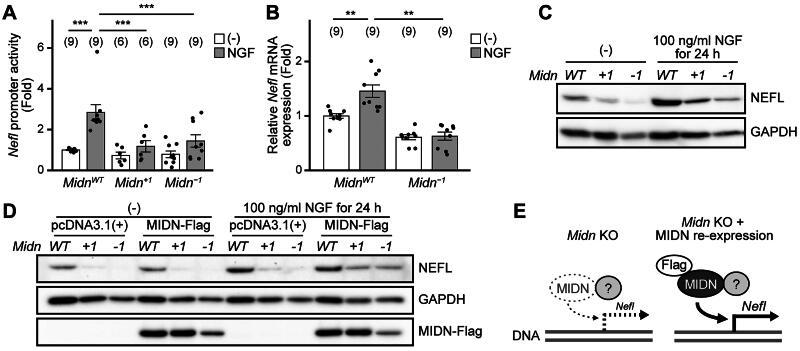
MIDN is involved in the expression of neurofilament light chain (NEFL) in PC12 cells. (A) *Nefl* promoter activity was measured as described in the Materials and Methods. PC12 cells were treated with or without NGF (100 ng/mL, 6 h). *Nefl* promoter activity was enhanced by NGF (100 ng/mL, 6 h) in *Midn*^WT^ PC12 cells; this effect was significantly attenuated in *Midn* KO PC12 cells. (B) Relative expression of *Nefl* mRNA, analyzed using quantitative real-time polymerase chain reaction (qPCR). PC12 cells were treated with NGF (100 ng/mL, 6 h). NGF-enhanced *Nefl* mRNA expression was significantly attenuated in *Midn* KO PC12 cells compared with that in *Midn*^WT^ PC12 cells. (C) Representative NEFL and glyceraldehyde-3-phosphate dehydrogenase (GAPDH) protein levels, measured by Western blotting. PC12 cells were treated with or without NGF (100 ng/mL, 24 h). NGF-enhanced NEFL protein levels were attenuated in *Midn* KO PC12 cells compared with those in *Midn*^WT^ PC12 cells. (D) Representative NEFL, GAPDH, and MIDN-Flag protein levels in PC12 cells transfected with either empty vector (pcDNA3.1[+]) or MIDN-Flag. Cells were treated with or without NGF (100 ng/mL, 24 h). Reduced NEFL expression in *Midn* KO cells treated with NGF was partially rescued by MIDN-Flag expression. (E) Schematic representation of the MIDN-Flag-dependent rescue of NEFL expression shown in (D). ***P < 0.001 (Tukey’s test). Data are expressed as the mean ± standard error.

Neurofilaments are neuron-specific intermediate filaments that function as major cytoskeletal proteins. They are also considered an index of neuronal differentiation.^23^ Of the neurofilament subunits, including neurofilament light chain (NEFL), neurofilament middle chain (NEFM), and neurofilament heavy chain (NEFH), *Nefl* is a known NGF stimulation-dependent gene. In addition, *Nefl* was the most highly expressed neurofilament in PC12 cells in our RNA-seq data (the reads per kb million [RPKM] of *Nefl*, *Nefm*, and *Nefh* were 109.84, 7.41, and 13.36, respectively). We therefore examined whether *Midn* is required for NEFL expression. We first explored *Nefl* promoter activity using a luciferase reporter plasmid.^24^ *Nefl* promoter activity was enhanced by NGF treatment (100 ng/mL, 6 h) in *Midn*^WT^ PC12 cells; this effect was significantly attenuated in *Midn* KO cells (Figure 2A). Similarly, both *Nefl* mRNA and NEFL protein levels were increased by NGF treatment (100 ng/mL, 6 and 24 h, respectively) in *Midn*^WT^ PC12 cells but were reduced in *Midn* KO cells at both basal and NGF-stimulated levels (Figure 2B and C). To confirm whether *Midn* regulates NEFL expression, Flag-tagged MIDN (MIDN-Flag) was overexpressed in *Midn* KO cells and NEFL expression was examined. In *Midn* KO cells, NEFL expression was significantly upregulated by MIDN-Flag expression compared with empty vector expression (*Midn^+1^*, 3.54-fold, *n* = 4, *P* = 0.021; *Midn^−1^*, 6.23-fold, *n* = 4, *P* = 0.021). The downregulation of NEFL protein levels in *Midn* KO cells was partially rescued by MIDN-Flag expression in the presence, but not the absence, of NGF (100 ng/mL, 24 h) (Figure 2D). These results suggest that MIDN regulates NGF-induced *Nefl* gene expression, and may help to explain why MIDN is responsible for neurite outgrowth. However, although MIDN regulates *Nefl* expression, MIDN itself does not contain a DNA-binding region. We therefore hypothesized that MIDN might associate with certain transcription factors and regulate their function (Figure 2E).

### MIDN binds directly to EGR1

**Figure 3.**
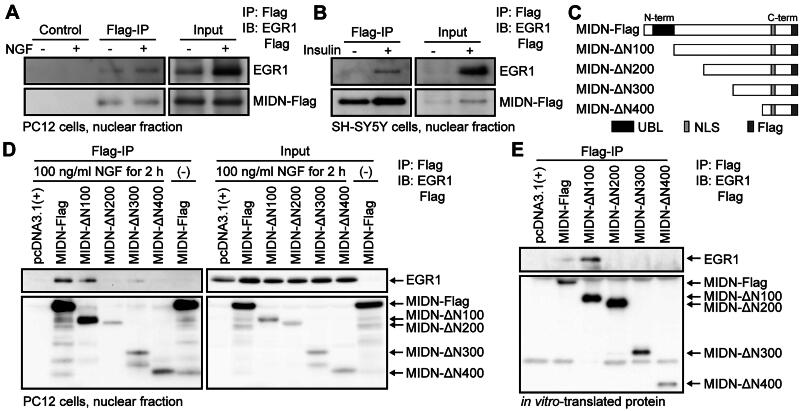
MIDN (101–200 amino acid [aa]) is required for direct binding to early growth response 1 (EGR1). (A) Representative EGR1 protein levels in PC12 cells co-immunoprecipitated with MIDN-Flag. PC12 cells were treated with or without NGF (100 ng/mL, 2 h). The nuclear fraction of the cells (Input), prepared as described in the Materials and Methods, was immunoprecipitated with sepharose beads (Control) or Flag-agarose beads (Flag-IP). EGR1 was co-immunoprecipitated with MIDN-Flag in PC12 cells. (B) Representative EGR1 protein levels in SH-SY5Y cells co-immunoprecipitated with MIDN-Flag. SH-SY5Y cells were treated with or without insulin (1 μM, 2 h). The nuclear fraction of the cells (Input) was precipitated with Flag-agarose beads (Flag-IP). EGR1 was co-immunoprecipitated with MIDN-Flag in SH-SY5Y cells. (C) Schematic representation of amino-terminal-truncated MIDN mutants, constructed as described in the Materials and Methods. UBL, ubiquitin-like domain; NLS, nuclear localization signal; N-term, amino-terminus; C-term, carboxy-terminus. (D) Representative EGR1 protein levels co-immunoprecipitated with MIDN mutants. PC12 cells were treated with or without NGF (100 ng/mL, 2 h). The nuclear fraction of cells transfected with either empty vector, MIDN-Flag, or Flag-tagged MIDN mutants (Input) was immunoprecipitated with Flag-agarose beads (Flag-IP). EGR1 was co-immunoprecipitated with MIDN-Flag and MIDN-ΔN100-Flag. (E) Representative EGR1 protein levels co-immunoprecipitated with MIDN mutants using in vitro-translated recombinant proteins, prepared as described in the Materials and Methods. MIDN (101–200 aa) was required for the direct binding to EGR1.

Previously, we performed a transcriptome analysis of *Midn*^WT^ and *Midn* KO polyclonal PC12 cells using RNA-seq.^20^ *Midn* KO affected the expression of a wide variety of genes. Notably, we identified that the consensus sequences of at least four transcription factors—EGR1 (probe accession number UP00007_1, *P* = 0.0018; UP00007_2, *P* = 0.0056), SMAD3 (UP00000_2, *P* = 0.0002, 0.0003, 0.0003, and 0.0023), GATA3 (UP00032_1, *P* = 0.0004), and PLAG1 (MA0163.1, *P* = 0.0043)—were shared in the promoter regions of downregulated genes in MIDN-deficient cells. Of these four factors, we focused on EGR1, an immediate–early gene, because EGR1 is known to be induced by NGF stimulation and is essential for neurite outgrowth in PC12 cells.^21^^,^^22^ In addition, although MIDN and EGR1 protein interaction has been reported^11^ or included in datasets of proteome analysis,^25^^,^^26^ EGR1 transcriptional activity has not yet been measured in these studies. In our previous transcriptome analysis, the RPKM values of EGR family proteins (EGR1, 2, 3, and 4) were 2.46, 0.10, 0.09, and 0.11, respectively, indicating that EGR1 is the most dominant EGR family protein in PC12 cells.^20^ In the present study, we therefore confirmed the association of MIDN with endogenous EGR1 in PC12 cells using immunoprecipitation. Specifically, the nuclear fraction of NGF-treated (100 ng/mL, 2 h) or -untreated PC12 cells was immunoprecipitated using control sepharose beads or Flag-agarose beads. EGR1, which is upregulated by NGF treatment, was co-immunoprecipitated with MIDN-Flag by Flag-agarose but not by control beads (Figure 3A). The association of MIDN-Flag with EGR1 in NGF-treated PC12 cells was also confirmed by our present liquid chromatography–tandem mass spectrometry (LC-MS/MS) analysis (Supplementary Table S1). In addition, the co-immunoprecipitation of MIDN-Flag with EGR1 was also detected in SH-SY5Y cells, a human neuroblastoma cell line, when treated with insulin (1 μM, 2 h) (Figure 3B). These findings thus confirm the interaction between MIDN and EGR1 in multiple cell lines.

To determine the MIDN regions required for its interaction with EGR1, we developed the following plasmids expressing MIDN mutants truncated at amino-terminal (N-terminal) amino acids (aa): MIDN-ΔN100, -ΔN200, -ΔN300, and -ΔN400 (Figure 3C). Of these truncated mutants, only MIDN-ΔN100 co-immunoprecipitated with EGR1 expressed in NGF-stimulated (100 ng/mL, 2 h) PC12 cells (Figure 3D). The expression levels of truncated MIDN mutants were much lower than those of full-length MIDN (Figure 3D, right), suggesting that the N-terminal region affects the stability of MIDN. To reduce the differences in expression levels of truncated MIDN mutants, we further confirmed the interaction between MIDN-ΔN100 and EGR1 using in vitro-translated protein (Figure 3E). Although the protein levels of MIDN-ΔN200 were higher than those of MIDN-ΔN100, in vitro-translated EGR1 was co-immunoprecipitated with in vitro-translated MIDN-ΔN100 but not with in vitro-translated MIDN-ΔN200. We confirmed the interaction between MIDN-ΔN100 and EGR1, and the interaction was assumed to be direct. These data suggest that MIDN (101–200 aa) is required for the association with EGR1. Consistent with these results, MIDN (101–200 aa) contains the Catch1 domain,^11^ which has been reported as a MIDN binding site for interacting proteins.

### MIDN is required for EGR1-dependent transcriptional activity

**Figure 4.**
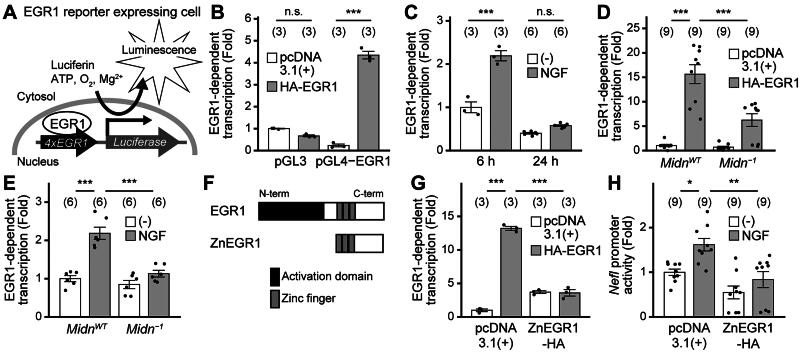
MIDN is required for EGR1 transcriptional activity. (A) Illustration of the detection of EGR1-dependent transcription using the pGL4-Egr1 (EGR1 reporter) plasmid. (B) EGR1-dependent transcription, measured as described in the Materials and Methods. PC12 cells transfected with either pGL3 empty vector or pGL4-Egr1 were cotransfected with either pcDNA3.1(+) empty vector or HA-tagged EGR1 (HA-EGR1). EGR1-dependent transcription was enhanced in PC12 cells transfected with both pGL4-Egr1 and HA-EGR1. (C) EGR1-dependent transcription that was enhanced by NGF treatment. PC12 cells transfected with pGL4-Egr1 were treated with or without NGF (100 ng/mL, either 6 h or 24 h). Treatment with NGF for 6 h enhanced EGR1-dependent transcription. (D) EGR1-dependent transcription in *Midn*^WT^ and *Midn*^−1^ PC12 cells. PC12 cells transfected with pGL4-Egr1 were cotransfected with either pcDNA3.1(+) empty vector or HA-EGR1. The EGR1-dependent transcription enhanced by HA-EGR1 expression was significantly attenuated in *Midn* KO PC12 cells compared with that in *Midn*^WT^ PC12 cells. (E) EGR1-dependent transcription that was enhanced by NGF treatment in *Midn*^WT^ and *Midn*^−1^ PC12 cells. PC12 cells transfected with pGL4-Egr1 were treated with or without NGF (100 ng/mL, 6 h). The EGR1-dependent transcription enhanced by NGF treatment was significantly attenuated in *Midn* KO PC12 cells compared with that in *Midn*^WT^ PC12 cells. (F) Schematic representation of ZnEGR1, a dominant-negative form of EGR1, constructed as described in the Materials and Methods. (G) EGR1-dependent transcription in PC12 cells transfected with either pcDNA3.1(+) empty vector or HA-tagged ZnEGR1 (ZnEGR1-HA). PC12 cells were cotransfected with pGL4-Egr1, pcDNA3.1(+) or HA-EGR1, and pcDNA3.1(+) or ZnEGR1-HA. The EGR1-dependent transcription enhanced by HA-EGR1 expression was significantly attenuated by ZnEGR1-HA expression. (H) *Nefl* promoter activity in PC12 cells transfected with either pcDNA3.1(+) empty vector or ZnEGR1-HA. PC12 cells transfected with *Nefl*-luciferase reporter were cotransfected with pcDNA3.1(+) or ZnEGR1-HA and treated with or without NGF (100 ng/mL, 6 h). The *Nefl* promoter activity enhanced by NGF treatment was significantly attenuated in ZnEGR1-expressing PC12 cells. n.s., not significant; *P < 0.05; **P < 0.01; ***P < 0.001 (Tukey’s test). Data are expressed as the mean ± standard error.

Although MIDN has been reported to enhance the degradation of transcription factors, including EGR1,^11^ EGR1 downstream genes were downregulated by *Midn* deficiency in our RNA-seq data.^20^ To address this discrepancy, we next attempted to examine EGR1 function. To investigate whether *Midn* regulates EGR1-dependent transcription, we developed a plasmid to monitor EGR1-dependent transcription (an EGR1 reporter). Four tandem EGR1 consensus sequences were inserted into the EGR1 reporter plasmid before the firefly luciferase cDNA sequence; the luciferase activity reflected EGR1-dependent transcription (Figure 4A). The reliability of the EGR1 reporter was confirmed by the response to EGR1 overexpression (Figure 4B) and NGF-dependent (100 ng/mL, 6 h) EGR1 expression (Figure 4C). We then examined whether *Midn* was required for EGR1 reporter activity. The EGR1-dependent transcription that was induced by both EGR1 overexpression and NGF treatment (100 ng/mL, 6 h) in *Midn*^WT^ PC12 cells was greatly reduced in *Midn^−1^* PC12 cells (Figure 4D and E).

We also examined the effects of EGR1 on *Nefl* expression by reducing EGR1 activity using a dominant-negative form of EGR1. It has been demonstrated that EGR1 activity is reduced by expressing a dominant-negative form of EGR1, ZnEGR1 (Figure 4F).^22^ ZnEGR1 is an EGR1 mutant lacking the transcriptional activation domain, and competes with EGR1 for DNA binding. To confirm the ZnEGR1-dependent reduction of EGR1 activity, we used the EGR1 reporter. As expected, we confirmed the ZnEGR1-dependent reduction of EGR1 reporter activity in EGR1-overexpressing PC12 cells (Figure 4G). Subsequently, we examined whether EGR1 inhibition affects *Nefl* expression. *Nefl* promoter activity was reduced by ZnEGR1 expression, suggesting that EGR1 is necessary for *Nefl* expression (Figure 4H).

### MIDN is required for EGR1 DNA binding

**Figure 5.**
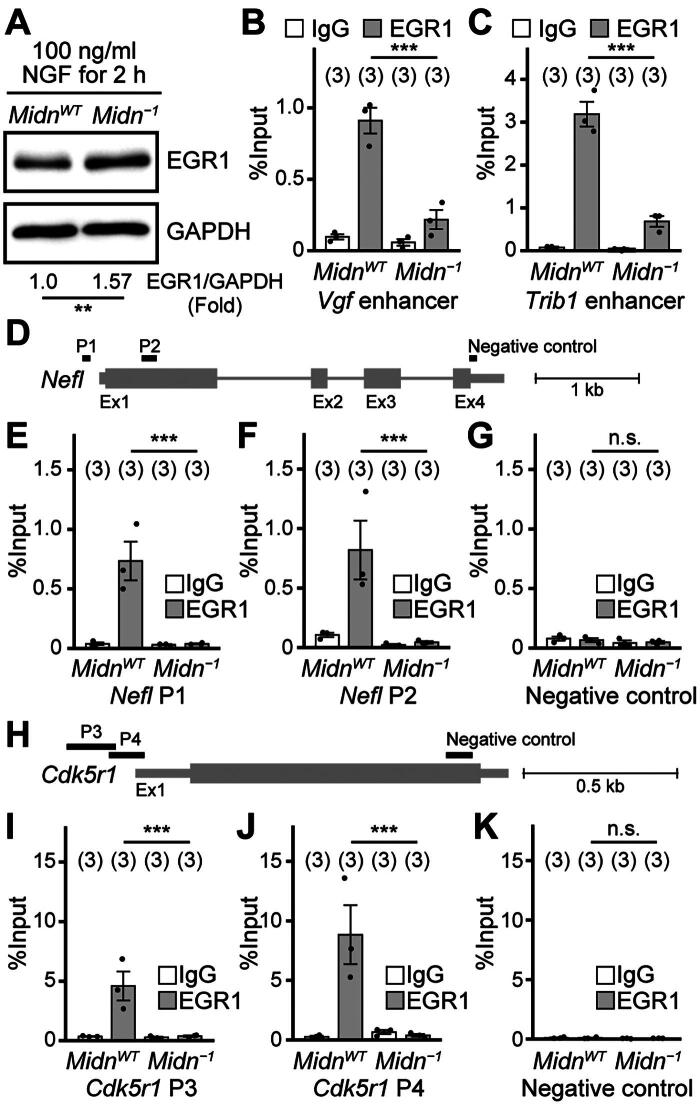
MIDN is required for EGR1 DNA binding in PC12 cells. (A) Representative EGR1 and GAPDH protein levels measured by Western blotting. PC12 cells were treated with NGF (100 ng/mL, 2 h). EGR1 expression levels normalized to GAPDH are indicated at the bottom. EGR1 protein levels were significantly increased in *Midn* KO PC12 cells compared with those in *Midn*^WT^ PC12 cells (*n* = 3). (B, C) Chromatin immunoprecipitation (ChIP)-qPCR assays using EGR1 antibody or mouse immunoglobulin G (IgG). Primers targeting the reported EGR1 binding sequences in VGF nerve growth factor inducible (*Vgf*) and tribbles pseudokinase 1 (*Trib1*) promoter were used as the positive control. (D) ChIP-qPCR primer design. The long line indicates the *Nefl* gene, and the short lines with P1, P2, and negative control indicate the amplified region by these primer sets used for the ChIP-qPCR analysis. Ex, exon. (E–G) ChIP-qPCR analysis in *Midn*^WT^ and *Midn* KO PC12 cells immunoprecipitated with either EGR1 antibody or control IgG. Primers shown in (D) were used. The EGR1 binding to the *Nefl* promoter region was significantly attenuated in *Midn* KO PC12 cells. (H) ChIP-qPCR primer design. The long line indicates the cyclin dependent kinase 5 regulatory subunit 1 (*Cdk5r1*) gene, and the short lines with P3, P4, and negative control indicate the amplified region by these primer sets used for the ChIP-qPCR analysis. (I–K) ChIP-qPCR analysis in *Midn*^WT^ and *Midn* KO PC12 cells immunoprecipitated with either EGR1 antibody or control IgG. Primers shown in (H) were used. The EGR1 binding to the *Cdk5r1* promoter region was significantly attenuated in *Midn* KO PC12 cells. *P* values were determined using Student’s *t* test (A) or Tukey’s test (B, C, E–G, I–K). n.s., not significant; **P < 0.01; ***P < 0.001. Data are expressed as the mean ± standard error.

We further examined the DNA binding of EGR1 using chromatin immunoprecipitation quantitative real-time polymerase chain reaction (ChIP-qPCR). We performed ChIP of EGR1 in *Midn*^WT^ and *Midn^−1^* PC12 cells treated with NGF (100 ng/mL, 2 h). Consistent with a previous report,^11^ the NGF-dependent expression of EGR1 was significantly increased in *Midn^−1^* PC12 cells (1.57-fold, *n* = 3, *P* = 0.005) (Figure 5A). The reliability of ChIP was confirmed by the binding of EGR1 to the reported EGR1 binding regions of the VGF nerve growth factor inducible (*Vgf*) promoter and tribbles pseudokinase 1 (*Trib1*) promoter^27^ (Figure 5B and C). Interestingly, EGR1 binding to the *Vgf* and *Trib1* promoter regions was inhibited by *Midn* KO (Figure 5B and C). Next, we examined EGR1 binding to *Nefl* promoter regions containing the EGR1 consensus sequences (primer sets 1 and 2 [P1 and P2]) and a negative control region (Figure 5D). EGR1 bound to P1 and P2 but not to the negative control (Figure 5E–G). However, the binding of EGR1 to P1 and P2 was completely inhibited in *Midn^−1^* PC12 cells, suggesting that *Midn* is required for the binding of EGR1 to *Nefl* promoter regions (Figure 5E and F). Furthermore, we examined EGR1 binding to the promoter regions of cyclin dependent kinase 5 regulatory subunit 1 (*Cdk5r1*, also known as *p35*), an EGR1 downstream gene that regulates neurite outgrowth.^21^ EGR1 binding to *Cdk5r1* promoter regions containing the EGR1 consensus sequences (P3 and P4) was completely inhibited in *Midn^−1^* PC12 cells (Figure 5H–K). Together, these findings suggest that MIDN is required for EGR1 DNA-binding activity even though EGR1 protein levels were significantly reduced by MIDN.

## Discussion

**Figure 6.**
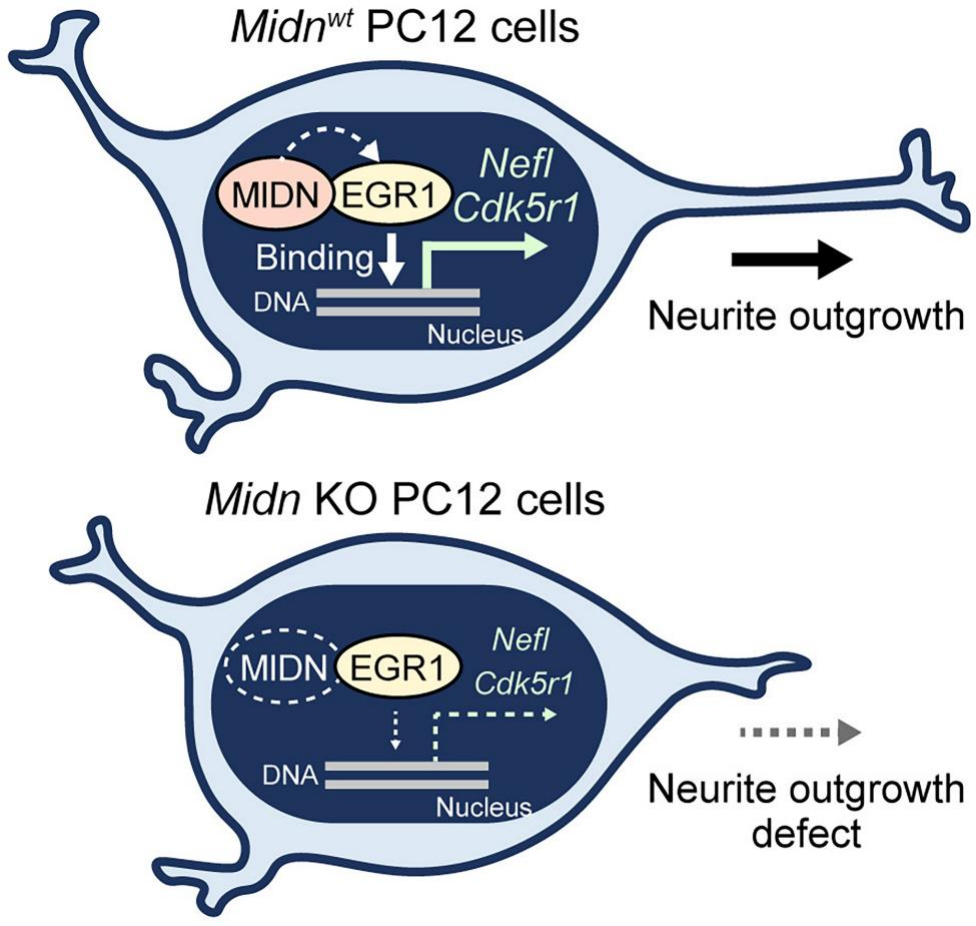
A putative schematic representation of the MIDN-dependent regulation mechanism of neurite outgrowth in PC12 cells. In *Midn*^WT^ PC12 cells (top), MIDN directly binds to EGR1 and enhances its DNA binding. As a result, the EGR1-dependent induction of *Nefl* and *Cdk5r1* promotes neurite outgrowth. In *Midn* KO PC12 cells (bottom), the reduced DNA binding of EGR1 inhibits *Nefl* and *Cdk5r1* expression, thus resulting in defective neurite outgrowth.

The present work focused on the function of MIDN in the regulation of neurite outgrowth. We first confirmed a direct interaction of MIDN and EGR1, which has been reported previously.^11^^,^^25^^,^^26^ In addition, we demonstrated that *Midn* is required for EGR1 binding to the promoter regions of *Nefl* and *Cdk5r1* (Figure 5), which are an index and a regulator of neurite outgrowth, respectively.^21^^,^^28^ EGR1 deficiency in PC12 cells leads to impaired neurite outgrowth,^22^ which is similar to the phenotype of *Midn* KO cells (Figure 1). In the current study, we revealed that reduced EGR1 transcriptional activity as a result of *Midn* KO is one of the reasons underlying the neurite outgrowth deficiency of *Midn* KO PC12 cells (Figure 6).

In our previous study, a reduction in *MIDN* copy number was demonstrated to increase the risk of PD.^9^^,^^18^ By contrast, there is reportedly no significant correlation between *MIDN* deletion and PD in the US;^29^ we consider that this discrepancy may be mainly caused by the different methodologies used in these studies.^30^ *Midn* deletion attenuates neurite outgrowth and PRKN ubiquitin ligase expression in PC12 cells.^9^ A reduction in activating transcription factor 4 (ATF4), a major regulator of *PRKN* expression,^31^ by *Midn* KO/knockdown in PC12 cells^9^ has also been confirmed in B cells from *Midn* knockdown mice.^17^ However, the molecular mechanism underlying the regulation by MIDN of PD risk remains unclear. In the present study, we demonstrated the MIDN-dependent regulation of EGR1 function (Figures 4 and 5); we presume that this mechanism is one of the potential regulatory mechanisms involved in the pathogenesis of PD. EGR1 is a member of the zinc finger family of transcription factors.^32^ In PC12 cells, EGR1 is required for neurite outgrowth.^22^ Neurites give rise to dendrites and axons; however, the function of EGR1 in animal models is to regulate synaptic plasticity, thus regulating learning and memory.^33^ In addition, the function of EGR1 in dopaminergic neurons remains controversial. EGR1 is reportedly inhibited by microRNA-181a-2-3p, which inhibits oxidative stress injury to dopaminergic neurons.^34^ Conversely, EGR1 has been reported as necessary for tyrosine hydroxylase expressing dopaminergic neurons of the zebrafish forebrain.^35^ Although the regulation of EGR1 by MIDN is likely one reason for the neurite outgrowth defects in *Midn* KO PC12 cells, a more detailed investigation into whether the regulation of EGR1 by MIDN is involved in PD pathogenesis is required.

Our LC-MS/MS analysis detected the binding of MIDN to proteasome subunits (Supplementary Table S1). This result supports the function of MIDN in enhancing ubiquitin-independent protein degradation by the proteasome^11^^,^^12^^,^^36^. Proteasomal degradation of unnecessary proteins, such as SNCA, slows down the progression of neurodegeneration in PD.^37^ In addition, proteasomal activity is required for axon formation in primary neurons.^38^ Disruption of MIDN-dependent protein degradation could lead to the disturbance of protein homeostasis and neurodegeneration. However, there might be a cell type-specific function mediated by proteasomal degradation, because neurite outgrowth in PC12 cells is reported to be enhanced by the treatment with the proteasome inhibitor.^39^ The relationship between proteasome function and *Midn* KO phenotype in neurons should be elucidated in future research.

Consistent with the reported function of MIDN in enhancing the degradation of immediate arly gene protein, such as EGR1,^11^^,^^36^ *Midn* KO increased EGR1 protein levels (Figure 5A). However, EGR1 functions such as DNA binding and transcriptional activity were robustly reduced by *Midn* KO (Figures 4 and 5). These results are consistent with the observed downregulation of genes with EGR1 binding sites in their promoter regions in our previous RNA-seq analysis.^20^ The regulatory mechanism underlying the discrepancy between EGR1 protein levels and function is an interesting research question. Although the MIDN-dependent proteasomal degradation pathway is assumed to be a relatively universal system, it is complex and many aspects remain unclear. Nonetheless, the MIDN-dependent proteasomal degradation of EGR1 inhibitory proteins is a putative mechanism regulating EGR1 function. EGR1 transcriptional activity is reportedly regulated by the transcriptional corepressors NGFI-A-binding proteins (NAB)1 and NAB2.^32^ However, the mRNA expression of *Nab1* and *Nab2* was not increased by *Midn* KO in the current study (data not shown), suggesting that these corepressors may not contribute much to MIDN-dependent EGR1 function. Alternatively, as another putative mechanism, the post-translational modification of EGR1 may be affected by MIDN. For example, EGR1 phosphorylation levels have been reported to regulate EGR1 DNA binding.^32^ Moreover, LC-MS/MS findings indicate that MIDN binds to several protein kinases.^11^ The regulation of phosphorylation may therefore be a candidate for the regulation of EGR1 by MIDN.

Recently, *Midn* loss-of-function models have been developed, and associations of MIDN with B cell leukemia malignancy and nonalcoholic fatty liver disease have been reported.^16^^,^^17^ However, the related phenotype in the brain has not yet been reported. Cell type-, tissue-, and substrate protein-specific phenomena likely exist, as demonstrated by the finding that proteasome activity in brain tissue is not altered in *Midn* knockdown mice, despite being decreased in other tissues.^17^ To clarify the role of MIDN in the brain, it will therefore be necessary to study the phenotypes of dopaminergic neurons in *Midn* KO animal models. Such investigations might reveal a novel mechanism of PD initiation, thus providing new targets for PD treatment. Further studies in both human patients and animal models are needed to better understand *MIDN* deficiency-dependent PD.

## Materials and Methods

### Materials

NGF (N0513), anti-Flag M2 affinity agarose gel (A2220), sepharose (4B200), Flag peptide (F3290), IGEPAL CA-630 (I8896), and Hoechst-33258 were purchased from Sigma-Aldrich (St Louis, MO). Recombinant human insulin (097-06474) was purchased from Wako (Osaka, Japan). Lipofectamine 2000 (52887) and rhodamine-phalloidin (R415) were purchased from Invitrogen (Grand Island, NY). Polyvinylidene difluoride (PVDF) membrane (10600023) was purchased from Cytiva (Buckinghamshire, UK). Antibodies against NEFL (#2835), Flag (#2368), EGR1 (#4154), and glyceraldehyde-3-phosphate dehydrogenase (GAPDH, #2118); horseradish peroxidase (HRP)-linked anti-GAPDH (#3683) antibody; HRP-linked anti-rabbit immunoglobulin G (IgG) secondary antibody (#7074); and HRP-linked anti-mouse IgG secondary antibody (#7076) were purchased from Cell Signaling Technology (Beverly, MA). Normal rabbit IgG (PM035) was purchased from MBL (Tokyo, Japan). Chemiluminescence substrate (NEL112001EA) was purchased from PerkinElmer (Waltham, MA). Dynabeads Protein G (DB10004) and Fast SYBR Green Master Mix (4385612) were purchased from Thermo Fisher Scientific (Waltham, MA). pGL3-basic vector (U47295), pGL4.34[luc2P SRF-RE Hygro] vector (E135A), and TNT^®^ Quick Coupled Transcription/Translation System (L1170) were purchased from Promega (Madison, WI). TriPure isolation reagent (11667165001) and a qPCR kit (06402712001) were purchased from Roche (Indianapolis, IN). A reverse transcription kit (TRT-101) and Can Get Signal Solution 1 (NKB-201) were purchased from Toyobo (Osaka, Japan). Micrococcal nuclease (M0247S) was purchased from New England Biolabs (Ipswich, MA). AMpure XP beads (A63880) were purchased from Beckman Coulter (Brea, CA).

### DNA constructs

The *Nefl*-luciferase reporter, consisting of a rat *Nefl* promoter and firefly luciferase cDNA, was kindly provided by Dr Heasley (University of Colorado Health Sciences Center, Denver, CO).^24^ A plasmid encoding β-actin promoter-driven β-galactosidase was kindly provided by Dr Philip Stork (Vollum Institute, Oregon Health Science University, Portland, OR).

For the measurement of EGR1 transcription, a pGL4-Egr1 plasmid was constructed as follows: the 4× EGR1 consensus sequence (annealed using the following oligonucleotide: 5′-CCG ACG CCC ACG CAC TCG ACG CCC ACG CAC TCG ACG CCC ACG CAC TCG ACG CCC ACG CAC TC-3′ and 5′-GAT CGA GTG CGT GGG CGT CGA GTG CGT GGG CGT CGA GTG CGT GGG CGT CGA GTG CGT GGG CGT CGG AGC T-3′; the EGR1 consensus sequences are underlined) was replaced with a serum response factor response element (SRF-RE) sequence of pGL4.34[luc2P SRF-RE Hygro] vector using SacI and BglII sites. DNA plasmids encoding carboxy-terminal Flag-tagged human *MIDN* (pcDNA3.1[+]-MIDN-Flag) and N-terminal hemagglutinin epitope-tagged (HA-tagged) human *EGR1* (pcDNA3.1[+]-HA-EGR1) were purchased from GenScript Japan (Tokyo, Japan). Deletion mutants of MIDN-Flag (*ΔN100*, *ΔN200*, *ΔN300*, and *ΔN400*) were generated using MIDN-Flag as a template, with a common reverse primer (5′-AAA GTC GAC TTA TCA CTT ATC GTC GTC ATC CTT G-3′) and the following forward primers: *ΔN100* (5′-CTT AAG CTT GCC ACC ATG GTG GAA GCG GGC CTC ATG TCT CAG G-3′), *ΔN200* (5′-CTT AAG CTT GCC ACC ATG CAG CAC GCT CCA CTG CAA CAC CGC C-3′), *ΔN300* (5′-CTT AAG CTT GCC ACC ATG GCC CCC CGC TCC CGA AAA CCC GGC G-3′), and *ΔN400* (5′-CTT AAG CTT GCC ACC ATG CCC TCA GCC TCC CTG CTG CAG GGC C-3′). Amplified *MIDN* mutant cDNA fragments were cut using HindIII and SalI and inserted into the multi-cloning site of the pcDNA3.1(+) vector using HindIII and XhoI sites. To construct pcDNA3.1(+)-EGR1-HA, the cDNA fragment encoding *EGR1* was inserted into the pcDNA3.1(+) vector using NheI and KpnI sites. Subsequently, the cDNA fragment encoding HA tag was inserted into the EGR1-inserted pcDNA3.1(+) vector using KpnI and XhoI sites. The *EGR1* fragment was amplified using pcDNA3.1(+)-HA-EGR1 as a template and the following primer sets: 5′-AAA GCT AGC GCC ACC ATG GCC GCG GCC AAG GCC GAG ATG C-3′ and 5′-TCA GGT ACC GCA AAT TTC AAT TGT CCT GGG AGA A-3′. The HA tag fragment was generated by annealing the following oligonucleotides: 5′-CTA CCC ATA CGA TGT TCC AGA TTA CGC TTA AC-3′ and 5′-TCG AGT TAA GCG TAA TCT GGA ACA TCG TAT GGG TAG GTA C-3′. For the expression of a dominant-negative EGR1 mutant (*ZnEGR1*),^22^ pcDNA3.1(+)-ZnEGR1-HA was generated. The cDNA fragment of *ZnEGR1* was generated using pcDNA3.1(+)-EGR1-HA as a template and the following primer set: 5′-AAA GCT AGC GCC ACC ATG CGC AAG TAC CCC AAC CGG CCC A-3′ and 5′-TCA GGT ACC CCG CAA GTG GAT CTT GGT ATG CCT C-3′. The *ZnEGR1* cDNA fragment was replaced with the EGR1 sequence in the pcDNA3.1(+)-EGR1-HA vector using NheI and KpnI sites.

### Cell culture

PC12 cells were grown in Dulbecco’s modified Eagle’s medium (DMEM) supplemented with 10% fetal bovine serum (FBS), 5% horse serum, penicillin (50 units/mL), and streptomycin (50 μg/mL) in a 5% CO_2_ incubator at 37 °C. *Midn* KO polyclonal PC12 cells^9^ were further cloned into monoclonal cells with a homozygous *Midn* frameshift mutation (+1/+1 insertion and -1/-1 deletion), as shown in Figure 1A. SH-SY5Y cells were cultured in DMEM supplemented with 10% fetal bovine serum and the aforementioned antibiotics. For the neurite outgrowth assay, after the cells were fixed with 4% paraformaldehyde/phosphate-buffered saline and treated with 0.5% TritonX-100, actin fibers and nuclei were stained with rhodamine-phalloidin (5 U/mL) and Hoechst-33258 (1 μg/mL), respectively. Photographs were taken using the CellInsight CX7 LZR High Content Analysis Platform (ThermoFisher Scientific KK, Tokyo, Japan), and total neurite length and the numbers of neurites and nuclei were measured using HCS Studio Neuronal Profiling BioApplication V4.2 (ThermoFisher Scientific KK) as described previously.^9^ Transfection into these cells was performed using Lipofectamine 2000.

### Luciferase assay

The luciferase reporter plasmids (e.g., *Nefl*-luciferase reporter, pGL3 vector, and pGL4-Egr1), β-actin promoter-driven β-galactosidase, and other plasmids (e.g., pcDNA3.1[+], pcDNA3.1[+]-HA-EGR1, and pcDNA3.1[+]-ZnEGR1-HA) were cotransfected into PC12 cells (1 × 10^5^ cells/well) using Lipofectamine 2000. Luciferase activity was measured as described previously.^10^ β-Galactosidase activity was measured to normalize the transfection efficiency.

### Immunoprecipitation

The nuclear fraction of PC12 cells was prepared according to the modified methods reported by Caspi et al.^40^ Briefly, cells cultured in a 10-cm dish were suspended in 500 μL of Buffer A (10 mM KCl, 0.1 mM ethylenediaminetetraacetic acid [EDTA], 0.1 mM ethylene glycol tetraacetic acid [EGTA], 1 mM dithiothreitol [DTT], 10 mM 4-(2-hydroxyethyl)-1-piperazineethanesulfonic acid [HEPES], pH 7.9) containing protease inhibitors and incubated on ice for 30 min. After the addition of IGEPAL CA-630 to reach a concentration of 3%, the samples were centrifuged (17,970 × *g* for 30 s), and the supernatant (cytoplasmic fraction) was removed. The pellet was resuspended in 300 μL of Buffer C (420 mM NaCl, 1 mM EDTA, 1 mM EGTA, 1 mM DTT, 20 mM HEPES, pH 7.9) containing protease inhibitors and incubated on ice for 30 min. After centrifugation (17,970 × *g* for 5 min), the supernatant was collected as the nuclear fraction. The lysates (500–1,000 μg/500 μL/tube) were precleared by incubating with sepharose beads (20 μL/tube) for 1 h at 4 °C. The supernatants were then incubated with either sepharose beads (20 μL/tube) or anti-Flag M2 affinity agarose gel (20 μL/tube) for 2 h at 4 °C. After washing the immunoprecipitates with the buffer, the MIDN-Flag complex was eluted by incubation with Flag peptide (0.5 mg/mL) for 45 min, and the supernatant was collected. MIDN-Flag, Flag-tagged MIDN mutants, and HA-EGR1 recombinant proteins were synthesized in vitro from their DNA plasmids using the TNT T7 Quick Coupled Transcription/Translation Systems according to the manufacturer’s instructions. The recombinant proteins were then mixed and used for immunoprecipitation.

### Western blotting

Samples lysed in Laemmli buffer were loaded and separated on 11% polyacrylamide gels by electrophoresis. Proteins were then transferred to PVDF membranes and blocked with 5% skim milk in Tris-buffered saline with 0.1% Tween-20 (TBST). Next, the blots were incubated with primary antibodies (1:1,000 dilution) overnight at 4 °C before being incubated with secondary antibodies (1:5,000 dilution) for 1.5 h at room temperature. Anti-EGR1 antibody was diluted in Can Get Signal Solution 1, and all other antibodies were diluted in 5% skim milk in TBST. The blots were developed using a chemiluminescent substrate and visualized with the ChemiDoc XRS imaging system (Bio-Rad, Hercules, CA). The relative band intensities (Figures 2D and 5A) were analyzed using Fiji.^41^

### qPCR

Cells were lysed and total RNA was isolated using TriPure isolation reagent. Total RNA (1 μg/tube) was reverse transcribed with Oligo-dT primers and qPCR was performed using a LightCycler Nano thermal cycler (Roche). The following primers were used: *Midn* (5′-GTG CCA CAC GCT GCA AAG-3′ and 5′-AAC TCG GAC TGG ATG TCT GG-3′), *Nefl* (5′-AGG ACA AGC AGA ATG CAG ACA T-3′ and 5′-GTA GCC GCT GGT TAT GCT ACC-3′), and ribosomal protein L27 (*Rpl27*) (5′-AAG CCG TCA TCG TAA AGA ACA-3′ and 5′-CTT GAT CTT GGA TCG CTT GGC-3′). The *Midn* primer used for cDNA sequencing was as follows: 5′-CCC CAA CTG CCA GGA TAG TA-3′ and 5′-AAC TCG GAC TGG ATG TCT GG-3′. *Nefl* and *Midn* expression was normalized to the *Rpl27* control and expressed as a fold change.

### ChIP assay

ChIP assays were performed as described previously^10^^,^^42^ with some modifications. After being cultured for 16 h in plain low-glucose DMEM, PC12 cells were treated with NGF (100 ng/mL, 2 h) in plain low-glucose DMEM. Ten million cells were then fixed and centrifuged. Cell pellets were suspended in nuclear extraction buffer before being centrifuged again. The resulting pellets were suspended in Buf NUC solution (15 mM HEPES-NaOH [pH 7.5], 60 mM KCl, 15 mM NaCl, 0.32 mM sucrose, 10 μg/mL aprotinin, 10 μg/mL leupeptin, 1 mM phenylmethylsulfonyl fluoride) mixed with calcium chloride, and were digested using micrococcal nuclease. After the addition of 2× sonication buffer to stop the reaction, the sample was centrifuged, and the supernatants were incubated with 10 μL anti-EGR1 antibody or 5 μg normal rabbit IgG control conjugated to magnetic beads. Bead-bound proteins were washed with buffer A (20 mM Tris-HCl [pH 8.0], 150 mM NaCl, 1 mM EDTA, 1% Triton X-100, 0.1% sodium deoxycholate, 0.1% sodium dodecyl sulfate [SDS]), buffer B (20 mM Tris-HCl [pH 8.0], 500 mM NaCl, 1 mM EDTA, 1% Triton X-100, 0.1% sodium deoxycholate, 0.1% SDS), buffer C (20 mM Tris-HCl [pH 8.0], 250 mM LiCl, 1 mM EDTA, 0.5% Nonidet P-40, 0.5% sodium deoxycholate), and TE buffer, and the samples were then eluted using RNase A, proteinase K, and sodium chloride. After a subsequent incubation with proteinase K, DNA was purified with AMpure XP beads, eluted with TE buffer, and subjected to real-time PCR analysis using Fast SYBR Green Master Mix. The primers for amplifying the *Nefl* promoter were 5′-CTT TGC TCT TGC GCA GAA TCC-3′ and 5′-CTG TGA TCG ATC GCG TTG C-3′ (*Nefl*, P1). Those of *Nefl* exon 1 were 5′-TGG AGC AGC AGA ACA AGG TC-3′ and 5′-GCT TCT CGT TAG TGG CGT CT-3′ (*Nefl*, P2). Those of *Nefl* exon 4 (the negative control region) were 5′-CCC ATT CCC AAC TAT CCC AGG-3′ and 5′-ACT CAA CTG GTT GGT TTG GTG A-3′ (*Nefl*, negative control). The primers for amplifying the *Cdk5r1* promoter were 5′-CAT TGC GGA GGG TCT GGG-3′ and 5′-CCC AGC CTA TGC TTC CAC TG-3′ (*Cdk5r1* amplicon 4, P3), or 5′-CAG TGG AAG CAT AGG CTG GG-3′ and 5′-ACA AGA TGG TTC TCA GCC CC-3′ (*Cdk5r1*, P4). Those of *Cdk5r1* exon 1 (the negative control region) were 5′-TGC AGA TCA ATG CTG ACC CA-3′ and 5′-GGA GTC GCT TCT TGT CCT CC-3′ (*Cdk5r1*, negative control). The primers for amplifying the *Vgf* and *Trib1* promoters have been reported by Adams et al.^27^

### Statistical analysis

Data are expressed as the mean ± standard error using ggplot2.^43^ The significance of any differences was analyzed using Student’s *t* test, Mann–Whitney U test, or two-way analysis of variance with post hoc Tukey’s test using R.

## Supplementary Material

Supplemental_material_R1_final.docx

Table_S1.pdf

## Data Availability

The LC-MS/MS data have been deposited to the ProteomeXchange Consortium via the jPOSTrepo^44^ with the dataset identifier PXD053265.

## Material Availability

There is a restriction on sharing the DNA plasmids of *Nefl*-luciferase reporter. Please contact Dr Lynn E. Heasley (University of Colorado Health Sciences Center, Denver, CO) for permission to obtain the DNA plasmid.
