# Supplementary material for: Midnolin, a Genetic Risk Factor for Parkinson’s Disease, Promotes Neurite Outgrowth Accompanied by Early Growth Response 1 Activation in PC12 Cells
Source: Mol Cell Biol. 2024 Sep 12;44(11):516–27. doi: 10.1080/10985549.2024.2399358 (PMC11529416; doi:10.1080/10985549.2024.2399358)
Supplement: Supplemental_material_R1_final.docx [file TMCB_A_2399358_SM5456.docx]

**Supplementary Material**

**Midnolin, a Genetic Risk Factor for Parkinson’s Disease, Promotes Neurite Outgrowth Accompanied by Early Growth Response 1 Activation in PC12 Cells**

Ayano Chiba^a^, Chisato Kato^a^, Tadashi Nakagawa^b^, Tsukasa Osaki^c^, Kohei Nakamura^a^, Ikuo Norota^a^, Mikako Nagashima^a^, Toru Hosoi^b^, Kuniaki Ishii^a^, Yutaro Obara^a^

*^a^Department of Pharmacology, Yamagata University School of Medicine, Yamagata, Japan*

*^b^Department of Clinical Pharmacology, Faculty of Pharmaceutical Sciences, Sanyo-Onoda City University, Sanyo Onoda, Japan*

*^c^Department of Biochemistry and Molecular Biology, Yamagata University School of Medicine, Yamagata, Japan*


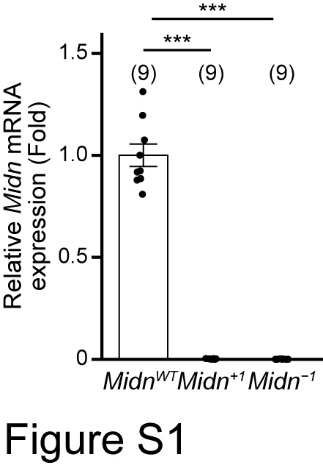


**Figure S1. Validation of midnolin (*Midn*) knockout (KO) PC12 cell lines**. Relative expression of *Midn* mRNA, analyzed using quantitative real-time polymerase chain reaction (qPCR). *Midn* mRNA expression was completely attenuated in *Midn* KO PC12 cell lines compared with that in wild-type (WT) PC12 cells. *Midn*^WT^, a WT clone; *Midn*^+1^, a single nucleotide-inserted clone; *Midn*^-1^, a single nucleotide-deleted clone. Each dot represents a single sample value; the number of samples is indicated at the top. ****p* < 0.001 (Tukey’s test). Data are expressed as the mean ± standard error.

**Supplementary Materials and Methods**

***Mass spectrometry.*** PC12 cells expressing Flag-tagged midnolin (MIDN-Flag) or pcDNA3.1(+) were treated with MG-132 (Sigma-Aldrich, St. Louis, MO; 5 μM, 6 h) and nerve growth factor (500 ng/mL, 2 h). The nuclear fractions of the cells were then immunoprecipitated with Flag-agarose beads. Co-immunoprecipitated proteins were reduced using dithiothreitol (10 mM) followed by alkylation with iodoacetamide (25 mM). Following hydrolysis with trypsin, the reaction mixtures were desalted using a C-Tip (Nikkyo Technos, Tokyo, Japan) as previously described^1^. The desalted peptide solution was analyzed by nanoflow liquid chromatography-tandem mass spectrometry (nLC-MS/MS) using the Dionex U3000 gradient pump (Thermo Fisher Scientific, Waltham, MA) coupled with a Q Exactive Hybrid Quadrupole-Orbitrap Mass Spectrometer (Thermo Fisher Scientific, Waltham, MA). Measurement conditions have been previously described^2^.

Raw file reads were matched against the Swiss-Prot database including rat and mouse (25,553 sequences), using Proteome Discoverer 2.4.1.15 (Thermo Fisher Scientific, Waltham, MA) with the Sequest^HT^ search engines. Precursor and fragment mass tolerances were set to 10 ppm and 0.02 Da, respectively. A fixed modification for S-carbamidomethylated cysteine, a variable modification for oxidized methionine, and two maximum missed cleavage sites for trypsin were set. The results were filtered using Percolator with a false discovery rate of 1%. The peak area of each identified peptide was estimated using Proteome Discoverer. The intensity of the unique peptide was used to calculate the protein intensity. The intensity-based absolute quantification (iBAQ) algorithm was used to calculate the protein quantification value^3^.

**References**

1. Osaki T, Sugiyama D, Magari Y, Souri M, Ichinose A. Rapid immunochromatographic test for detection of anti-factor XIII A subunit antibodies can diagnose 90 % of cases with autoimmune haemorrhaphilia XIII/13. *J Thromb Haemost.* 2015;13(5):802-814. doi: 10.1111/jth.12877.
2. Fujino T, Sonoda R, Higashinagata T, Mishiro-Sato E, Kano K, Murakami H. Ser/Leu-swapped cell-free translation system constructed with natural/in vitro transcribed-hybrid tRNA set. *Nat Commun*. 2024;15(1):4143. doi: 10.1038/s41467-024-48056-z.
3. Schwanhäusser B, Busse D, Li N, Dittmar G, Schuchhardt J, Wolf J, Chen W, Selbach M. Global quantification of mammalian gene expression control. *Nature* 2011;19:473(7347):337-342. doi: 10.1038/nature10098.
