# Supplementary material for: Midnolin, a Genetic Risk Factor for Parkinson’s Disease, Promotes Neurite Outgrowth Accompanied by Early Growth Response 1 Activation in PC12 Cells
Source: Mol Cell Biol. 2024 Sep 12;44(11):516–27. doi: 10.1080/10985549.2024.2399358 (PMC11529416; doi:10.1080/10985549.2024.2399358)
Supplement: Table_S1.pdf [file TMCB_A_2399358_SM5455.pdf]

Supplementary Table S1. Proteomic analysis of MIDN-binding proteins in PC12 cells.

A	B	C	D	E	F	G	H	I	J	K	L	M
Accession	Description	Species	Gene Symbol	Coverage [%]	# Peptides	# PSMs	# AAs	MW [kDa]	calc. pI	Abundance: cont	Abundance: MIDN	Abundance Ratio (log2): (MIDN) / (cont)
Q504T8	Midnolin	Homo sapiens	MIDN	75	20	130	468	49.2	9.55	41616.59375	175297602	12.04
P08154	Early growth response protein 1	Rattus norvegicus	Egr1	15	6	15	508	53.9	8.41	39460.78516	6660565.9	7.40
P62198	26S proteasome regulatory subunit 8	Rattus norvegicus	Psmc5	40	13	26	406	45.6	7.55	288731.7676	11281254	5.29
O88761	26S proteasome non-ATPase regulatory subunit 1	Rattus norvegicus	Psmd1	31	20	34	953	105.7	5.39	543685.1934	17552593	5.01
P40307	Proteasome subunit beta type-2	Rattus norvegicus	Psmb2	57	7	13	201	22.9	7.44	327633.9844	6181713.7	4.24
P17220	Proteasome subunit alpha type-2	Rattus norvegicus	Psma2	20	3	4	234	25.9	7.43	91323.35156	1488005.3	4.03
Q63570	26S proteasome regulatory subunit 6B	Rattus norvegicus	Psmc4	47	17	36	418	47.4	5.21	808227.4219	12996169	4.01
P28073	Proteasome subunit beta type-6	Rattus norvegicus	Psmb6	18	4	5	238	25.3	5	138816.8672	1966014.3	3.82
P48004	Proteasome subunit alpha type-7	Rattus norvegicus	Psma7	36	7	13	254	28.3	8.68	345636.0332	3939368.1	3.51
B0BN93	26S proteasome non-ATPase regulatory subunit 13	Rattus norvegicus	Psmd13	13	4	5	376	42.8	5.83	166122.8828	1568371.3	3.24
P18420	Proteasome subunit alpha type-1	Rattus norvegicus	Psma1	28	5	5	263	29.5	6.61	248785.4688	2294828.2	3.21
P07153	Dolichyl-diphosphooligosaccharide--protein glycosyltransferase subunit 1	Rattus norvegicus	Rpn1	13	5	5	605	68.3	6.51	141866.125	824177.92	2.54
O08623	Sequestosome-1	Rattus norvegicus	Sqstm1	12	3	7	439	47.7	5.17	279511.9355	1597550.7	2.51
P42930	Heat shock protein beta-1	Rattus norvegicus	Hspb1	55	9	21	206	22.9	6.55	1697294.432	9125913.8	2.43
P19944	Large ribosomal subunit protein P1	Rattus norvegicus	Rplp1	52	2	4	114	11.5	4.32	335239.1797	1424581.2	2.09
P0DMW0	Heat shock 70 kDa protein 1A	Rattus norvegicus	Hspa1a	36	15	34	641	70.1	5.82	2523615.113	9547263.9	1.92
P04177	Tyrosine 3-monooxygenase	Rattus norvegicus	Th	27	8	9	498	55.9	6.11	1116657.031	4205213.3	1.91
P69897	Tubulin beta-5 chain	Rattus norvegicus	Tubb5	68	19	60	444	49.6	4.89	10111137.35	32794349	1.70
P63018	Heat shock cognate 71 kDa protein	Rattus norvegicus	Hspa8	42	21	69	646	70.8	5.52	14183723.13	45707548	1.69
P62282	Small ribosomal subunit protein uS17	Rattus norvegicus	Rps11	35	5	11	158	18.4	10.3	517983.9219	1647645	1.67
O08629	Transcription intermediary factor 1-beta	Rattus norvegicus	Trim28	7	5	7	835	88.9	5.77	1021786.25	3225192.7	1.66
P13383	Nucleolin	Rattus norvegicus	Ncl	13	8	13	713	77.1	4.74	1477722.047	4116623.6	1.48
P21807	Peripherin	Rattus norvegicus	Prph	59	23	54	468	53.5	5.47	8642419.754	24001799	1.47
P38650	Cytoplasmic dynein 1 heavy chain 1	Rattus norvegicus	Dync1h1	18	65	101	4644	531.9	6.46	169260.3281	441835.56	1.38
Q99JD4	CLIP-associating protein 2	Rattus norvegicus	Clasp2	30	31	58	1286	140.6	8.57	8570993.469	20516054	1.26
P62909	Small ribosomal subunit protein uS3	Rattus norvegicus	Rps3	20	4	4	243	26.7	9.66	593502.3281	1394145.5	1.23
Q05962	ADP/ATP translocase 1	Rattus norvegicus	Slc25a4	20	5	7	298	33	9.79	1609372.586	3673187.3	1.19
Q62910	Synaptojanin-1	Rattus norvegicus	Synj1	10	13	21	1574	172.8	6.87	5268397.578	11969132	1.18
P62832	Large ribosomal subunit protein uL14	Rattus norvegicus	Rpl23	27	3	7	140	14.9	10.51	880080.6719	1967832	1.16
O35821	Myb-binding protein 1A	Rattus norvegicus	Mybbp1a	10	11	19	1344	152.2	8.95	1678091.422	3741473.5	1.16
P62828	GTP-binding nuclear protein Ran	Rattus norvegicus	Ran	33	6	15	216	24.4	7.49	2075973.586	4614534.1	1.15
Q6AYH5	Dynactin subunit 2	Rattus norvegicus	Dctn2	12	4	5	402	44.1	5.26	731506.25	1625357.8	1.15
Q62826	Heterogeneous nuclear ribonucleoprotein M	Rattus norvegicus	Hnrnpm	10	7	9	690	73.7	8.75	1100448.82	2413473.7	1.13
P61980	Heterogeneous nuclear ribonucleoprotein K	Rattus norvegicus	Hnrnpk	46	19	104	463	50.9	5.54	38997218.75	80391070	1.04
P62703	Small ribosomal subunit protein eS4	Rattus norvegicus	Rps4x	12	3	9	263	29.6	10.15	644770.9609	1320518	1.03
P48679	Prelamin-A/C	Rattus norvegicus	Lmna	14	8	20	665	74.3	6.98	1445602.531	2940158.9	1.02

Human midnolin (MIDN) and *Rattus norvegicus*- derived proteins (column C) with abundance ratios in the MIDN-binding fraction (column L) relative to the control (column K) greater than 2 (column M greater than 1.0) are listed. They were also identified by multiple peptides (column F).
